# Tegmental atrophy in isolated REM sleep behaviour disorder: *ex vivo* MRI–informed *in vivo* imaging

**DOI:** 10.1093/braincomms/fcag379

**Published:** 2026-10-08

**Authors:** Masakazu Hirose, Kenji Yoshinaga, Yoshifumi Mori, Atsushi Shima, Nobukatsu Sawamoto, Noritaka Wakasugi, Hirohiko Imai, Masahiko Watanabe, Goichi Beck, Yuta Kajiyama, Ujiakira Nishiike, Hideki Mochizuki, Kazuya Kawabata, Keita Hiraga, Tomohiko Nakamura, Masahisa Katsuno, Hirohisa Watanabe, Taku Hatano, Koji Kamagata, Nobutaka Hattori, Akira Nishida, Yohei Mukai, Eiko N Minakawa, Yuji Takahashi, Ryosuke Takahashi, Takashi Hanakawa

**Affiliations:** Department of Integrated Neuroanatomy and Neuroimaging, Kyoto University Graduate School of Medicine, Kyoto 606-8501, Japan; Department of Neurology, Kyoto University Graduate School of Medicine, Kyoto 606-8507, Japan; Department of Integrated Neuroanatomy and Neuroimaging, Kyoto University Graduate School of Medicine, Kyoto 606-8501, Japan; Department of Integrated Neuroanatomy and Neuroimaging, Kyoto University Graduate School of Medicine, Kyoto 606-8501, Japan; Computational Cognitive Neuroscience & Neuroimaging, Netherlands Institute for Neuroscience, Royal Netherlands Academy of Arts and Sciences, 1105 BA Amsterdam, the Netherlands; Department of Neurology, Kyoto University Graduate School of Medicine, Kyoto 606-8507, Japan; Human Brain Research Centre, Kyoto University Graduate School of Medicine, Kyoto 606-8507, Japan; Department of Human Health Sciences, Kyoto University Graduate School of Medicine, Kyoto 606-8507, Japan; Department of Human Health Sciences, Kyoto University Graduate School of Medicine, Kyoto 606-8507, Japan; Department of Advanced Neuroimaging, Integrative Brain Imaging Center, National Center Hospital, National Center of Neurology and Psychiatry, Tokyo 187-8551, Japan; Innovation Research Centre for Quantum Medicine, Gifu University School of Medicine, Gifu 501-1194, Japan; Department of Anatomy, Faculty of Medicine, Hokkaido University, Sapporo, Hokkaido 060-8638, Japan; Department of Neurology, Graduate School of Medicine, University of Osaka, Suita, Osaka 565-0871, Japan; Department of Neurology, Graduate School of Medicine, University of Osaka, Suita, Osaka 565-0871, Japan; Department of Neurology, Graduate School of Medicine, University of Osaka, Suita, Osaka 565-0871, Japan; Department of Neurology, Graduate School of Medicine, University of Osaka, Suita, Osaka 565-0871, Japan; Department of Neurology, Fujita Health University, Toyoake, Aichi 470-1192, Japan; Division of BrainTheraInformatics, International Center for Brain Science, Fujita Health University, Toyoake, Aichi 470-1192, Japan; Department of Neurology, Nagoya University Graduate School of Medicine, Nagoya, Aichi 466-8550, Japan; Department of Neurology, Nagoya University Graduate School of Medicine, Nagoya, Aichi 466-8550, Japan; Department of Neurology, Hamamatsu University Hospital, Hamamatsu, Shizuoka 431-3192, Japan; Department of Neurology, Nagoya University Graduate School of Medicine, Nagoya, Aichi 466-8550, Japan; Department of Neurology, Fujita Health University, Toyoake, Aichi 470-1192, Japan; Department of Neurology, Faculty of Medicine, Juntendo University, Tokyo 113-8421, Japan; Department of Radiology, Juntendo University Graduate School of Medicine, Tokyo 113-8421, Japan; Department of Neurology, Faculty of Medicine, Juntendo University, Tokyo 113-8421, Japan; Department of Neurology, Kyoto University Graduate School of Medicine, Kyoto 606-8507, Japan; Department of Neurology, Kurashiki Central Hospital, Kurashiki, Okayama 710-8602, Japan; Department of Neurology, National Center Hospital, National Center of Neurology and Psychiatry, Kodaira, Tokyo 187-8551, Japan; Department of Neurophysiology, National Institute of Neuroscience, National Center of Neurology and Psychiatry, Kodaira, Tokyo 187-8502, Japan; Department of Neurology, National Center Hospital, National Center of Neurology and Psychiatry, Kodaira, Tokyo 187-8551, Japan; Department of Neurology, Kyoto University Graduate School of Medicine, Kyoto 606-8507, Japan; Department of Integrated Neuroanatomy and Neuroimaging, Kyoto University Graduate School of Medicine, Kyoto 606-8501, Japan; Human Brain Research Centre, Kyoto University Graduate School of Medicine, Kyoto 606-8507, Japan; Department of Advanced Neuroimaging, Integrative Brain Imaging Center, National Center Hospital, National Center of Neurology and Psychiatry, Tokyo 187-8551, Japan

**Keywords:** REM sleep behaviour disorder, laterodorsal tegmental nucleus ventral part, corticotropin-releasing hormone binding protein, probabilistic atlas, alpha-synucleinopathy

## Abstract

Isolated rapid eye movement (REM) sleep behaviour disorder (iRBD) is an early-stage synucleinopathy characterized by brainstem pathology. In rodents, the pontine tegmentum contains a REM sleep centre, the sublaterodorsal nucleus, which expresses corticotropin-releasing hormone binding protein (CRHBP). While the involvement of brainstem pathophysiology is thus implicated in iRBD, its solid evidence remains scarce in humans due to the difficulty in identifying small brainstem nuclei with conventional MRI technology alone. Here, we aimed to detect tegmental atrophy in iRBD with voxel-based morphometry (VBM) analysis combined with a novel human brainstem atlas. Structural MRIs from 98 patients with iRBD and 114 controls were analysed to investigate grey matter volume using VBM. Our unique approach involved detailed assessments of the VBM results, guided by a high-resolution MRI-based atlas of the human brainstem. This brainstem atlas was founded on *ex vivo* MRI of 10 post-mortem human specimens. We validated it with CRHBP immunostaining, which aided in identifying putative REM sleep-regulating nuclei in humans. We applied this brainstem atlas to identify atrophy in specific brainstem regions in iRBD and correlate their volumes with clinical and polysomnographic measures. Voxel-based morphometry revealed a focal cluster of grey matter atrophy in the dorsal pontine tegmentum of iRBD patients, including the laterodorsal tegmental nucleus, ventral part (LDTgV) and the pedunculopontine tegmental nucleus (PTg). Our atlas-based analysis confirmed the LDTgV as the site of most conspicuous atrophy, revealing a significant volume reduction in iRBD patients compared with controls with a moderate effect size (Cohen’s *d* = 0.44, Bonferroni-corrected *P* = 0.019). Furthermore, greater atrophy in the LDTgV and the PTg was associated with more severe autonomic dysfunction as measured by Scales for Outcomes in Parkinson’s Disease-Autonomic dysfunction scores (partial *r* = −0.237, *P* = 0.021 and partial *r* = −0.236, *P* = 0.021, respectively). In 23 patients from whom quantitative polysomnographic data were available, LDTgV atrophy was correlated with the percentage ratio of REM sleep without atonia (partial *r* = −0.491, *P* = 0.028). Histological analysis confirmed that the LDTgV is selectively enriched with CRHBP-positive neurons, a putative marker for REM sleep-on neurons. We provided novel evidence for the involvement of LDTgV, the putative human homologue of the murine sublaterodorsal nucleus, in iRBD. The present findings advance our understanding of the neuroanatomical basis of iRBD and will contribute to the development of early biomarkers for α-synucleinopathies.

## Graphical Abstract

**Graphical Abstract:**
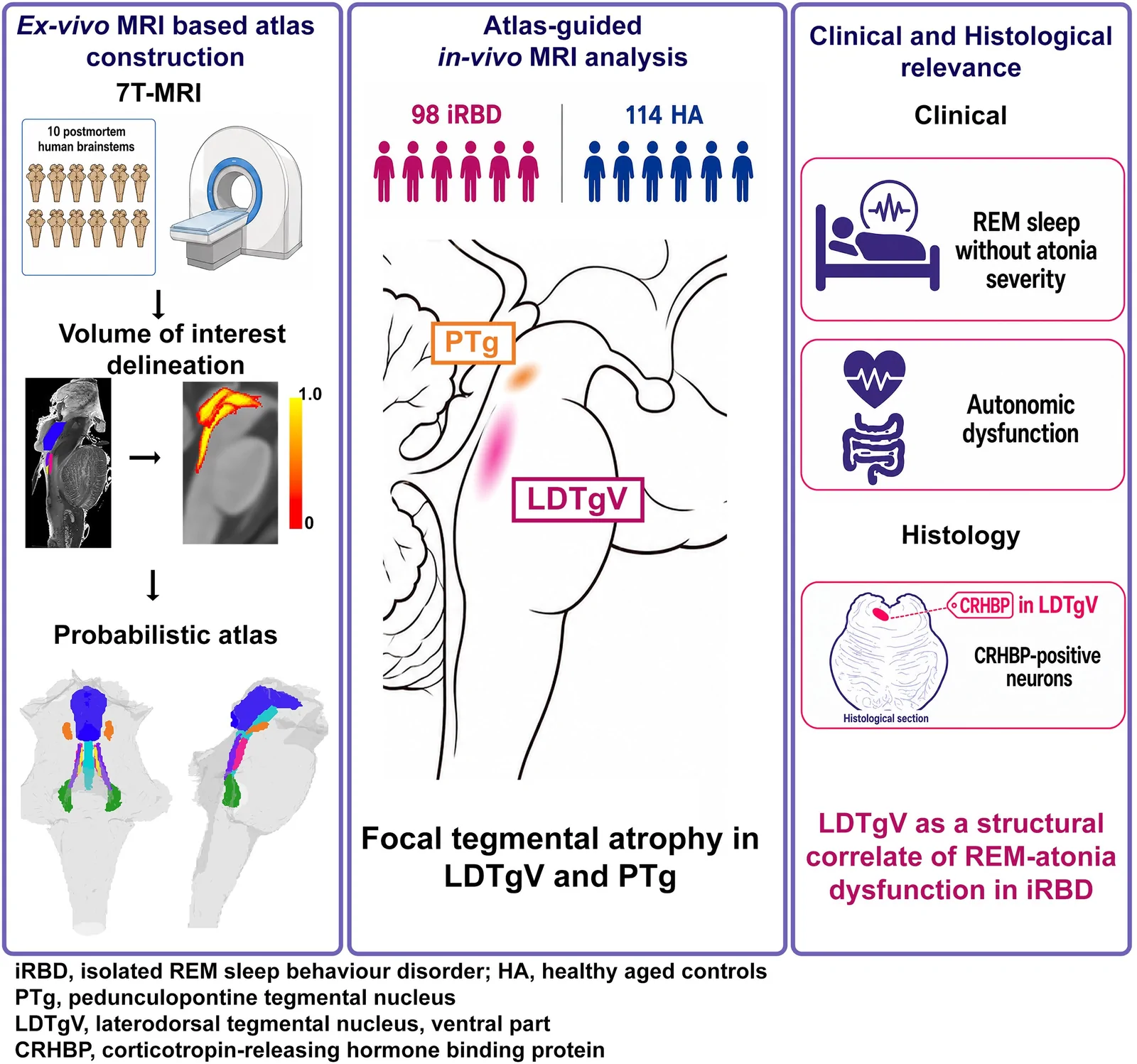

## Teaser

Hirose *et al*. report that a histologically validated brainstem atlas, built from high-resolution MRI of post-mortem human specimens, reveals selective atrophy of a small pontine tegmental nucleus in patients with isolated rapid eye movement sleep behaviour disorder. This bridges post-mortem and living-brain imaging, enabling early biomarkers of neurodegenerative disease.

## Introduction

Isolated rapid eye movement (REM) sleep behaviour disorder (iRBD) is characterized by dream-enacting behaviours and REM sleep without atonia. Cohort studies of iRBD have shown that 60–76% of patients develop α-synucleinopathy—Parkinson’s disease, dementia with Lewy bodies or multiple system atrophy—within 10 years after diagnosis.^1-3^ This high conversion rate has led iRBD to be widely recognized as one of the strongest prodromal markers of α-synucleinopathy.^4,5^ A common and salient feature of this prodromal phase is early autonomic dysfunction, often preceding motor and cognitive decline and predicting conversion to α-synucleinopathy.^1,6-8^ Nevertheless, the specific neurodegenerative processes underlying iRBD and its associated features remain unclear.

The brainstem, particularly the ponto-mesencephalic tegmentum, plays a pivotal role in regulating REM sleep, as inferred from animal models.^9-11^ This region controls sleep states through a ‘flip-flop’ mechanism involving neurons that are specifically active during REM sleep (REM-on) and those that suppress REM sleep (REM-off).^12,13^ In rodents, these REM-on neurons are identified in the sublaterodorsal tegmental nucleus (SLD), the pedunculopontine tegmental nucleus (PTg) and the laterodorsal tegmental nucleus (LDT). These neurons interact with medulla oblongata neurons, regulating REM sleep and atonia. Among these, the SLD, also known as the subLDT,^14^ appears to be the critical region in regulating REM sleep. A recent study has identified glutamatergic neurons expressing corticotropin-releasing hormone binding protein (CRHBP) in the murine SLD, and these neurons are crucial in generating muscle atonia during REM sleep.^15^ Notably, in post-mortem investigations of human patients with Parkinson’s disease and RBD, the CRHBP-immunoreactive neurons in the pontine tegmentum contained pathological α-synuclein.^15^ This seminal study for the first time connected the pontine tegmentum and the pathophysiology of RBD in both rodents and humans.

However, translating these fundamental neuroanatomical insights into neuroimaging markers from living human patients remains a substantial challenge. Although previous *in vivo* structural MRI studies have reported alterations in grey matter volume (GMV) in iRBD using voxel-based morphometry (VBM), findings have been inconsistent concerning the brainstem.^16-18^ Indeed, although a recent VBM meta-analysis consistently found GMV changes in some cortical and subcortical regions, it failed to identify a uniform pattern of GMV changes within the brainstem.^19^ Beyond these inter-study discrepancies, a hurdle is the difficulty in precisely identifying specific brainstem nuclei *in vivo*. For instance, while neuromelanin MRI enables the visualization of a small nucleus, such as the locus coeruleus (LC),^20-24^ the precise localization of other nuclei remains challenging.

Most importantly, the human homologue of the SLD remains under debate, with candidates including the subcoeruleus nucleus (SubC) and the ventral subdivisions of the LDT (LDTgV).^14,25,26^ Resolving this debate requires the precise differentiation of these candidate nuclei from adjacent structures, including the separation between the dorsal (i.e. LDTg) and ventral (i.e. LDTgV) parts of the LDT complex, a task not feasible with conventional approaches. A comprehensive, high-resolution, histologically validated brainstem atlas is essential for enhancing our understanding of the neuroanatomical basis of iRBD and its relationship to α-synucleinopathy progression.

Here, to address brainstem abnormalities in iRBD, we combined an in-house, ultra-high-resolution atlas of the human brainstem with structural MRI analyses of 98 iRBD patients and 114 matched controls from a multi-centre cohort. We examined 10 post-mortem human brainstems using *ex vivo* 7-T MRI, visualizing the overlap of small brainstem nuclei across the 10 specimens in the standard MRI space. We validated this probabilistic brainstem atlas with histology, including CRHBP immunohistochemistry (IHC). Our primary hypothesis was that this atlas-assisted approach would reveal atrophy of specific brainstem nuclei, corresponding to the putative human homologue of the SLD, which would be overlooked with conventional MRI techniques alone. Considering the prominence of autonomic dysfunction as an early feature of iRBD, we further hypothesized that structural alterations within key brainstem regions implicated in iRBD pathophysiology would correlate with the severity of autonomic symptoms. In a subset of 23 patients from whom quantitative polysomnography (PSG) data were available, we examined whether regional GMV was associated with the proportion of REM sleep without atonia. Integrating high-resolution *ex vivo* mapping and *in vivo* structural MRI analysis allowed us to identify brainstem markers of prodromal α-synucleinopathy in humans and their clinical relevance.

## Materials and methods

### *In vivo* MRI analysis

#### Cohort participants and data acquisition

We analysed data from 98 patients with PSG-confirmed iRBD and 114 healthy aged people. The patients with iRBD participated in the Japan Parkinson’s Progression Markers Initiative (J-PPMI) at five hospitals in Japan: The National Center of Neurology and Psychiatry (NCNP) Hospital (*n* = 54), Juntendo University Hospital (*n* = 20), Kyoto University Hospital (*n* = 12), The University of Osaka Hospital (*n* = 4) and Nagoya University Hospital (*n* = 8). A detailed description of the J-PPMI cohort has been previously published.^27^ In brief, the J-PPMI cohort recruited males and females aged 60 years or older diagnosed with iRBD. The diagnosis of iRBD followed the International Classification of Sleep Disorders criteria (3rd revision),^28^ including confirmation of REM sleep without atonia using PSG. Exclusion criteria included neurological or psychiatric disorders other than iRBD, a Geriatric Depression Scale-15 score of 10 or higher, or a State-Trait Anxiety Inventory Form Y-1 score of 65 or higher. The healthy aged control group (HA) consisted of individuals recruited from the NCNP Hospital (*n* = 83) and Kyoto University Hospital (*n* = 31). Participants in the HA group had no history of neurological or psychiatric disorders, as confirmed by clinical interview and a screening questionnaire originally for MRI scanning. However, PSG or REM Sleep Behavior Disorder Screening Questionnaire-Japanese (RBDSQ-J) data were unavailable from the HA group. The study protocol was approved by the local ethics committees (A2014-127 in NCNP, 16-154 in Juntendo, C1059-6 in Kyoto, 14454 in Osaka and 2015-0169 in Nagoya).

The patients with iRBD completed a battery of clinical evaluations: The Japanese version of the Montreal Cognitive Assessment (MoCA-J) for cognitive function, Scales for Outcomes in Parkinson’s Disease-Autonomic Dysfunction (SCOPA-AUT) for autonomic symptoms, Movement Disorder Society-Sponsored Revision of the Unified Parkinson’s Disease Rating Scale (MDS-UPDRS) for motor symptoms and RBDSQ-J for screening RBD symptoms. For evaluating cognitive functions in the control HA group, the Mini-Mental State Examination score was available, which was subsequently converted to estimated MoCA scores using a proposed algorithm.^29^ All participants underwent 3-dimensional (3D) T1-weighted structural MRI scanning at a magnetic field strength of 3 T. The imaging protocol was standardized across all sites, primarily using a magnetization-prepared rapid acquisition with gradient echo sequence with an isotropic resolution of 1 mm (Supplementary Table 1). Scanning parameters were optimized for each scanner while maintaining comparable image quality.

#### VBM analysis

We performed image processing and VBM analysis with SPM12 (https://www.fil.ion.ucl.ac.uk/spm/software/spm12/) implemented on MATLAB R2022 (MathWorks, Natick, MA, USA). The processing pipeline included bias field correction and tissue-class segmentation using the standard tissue probability maps. Spatial normalization to the Montreal Neurological Institute (MNI152) space utilized a template for East Asian brains, with the diffeomorphic anatomical registration through the exponentiated Lie algebra algorithm. Jacobian modulation was applied to GMV images to preserve absolute tissue volumes, followed by smoothing with a 6-mm full-width at half-maximum Gaussian kernel. To mitigate the effects of different MRI scanners, ComBat harmonization was applied at each voxel of the spatially normalized GMV images, modelling age, sex, total intracranial volume (TIV) and disease label as covariates of interest, while accounting for site effects. The harmonization procedure was validated by confirming a substantial reduction in between-site variance after the ComBat correction (see Supplementary Fig. 1, Supplementary Table 2). The site-harmonized GMV images were fed into VBM analysis with age, sex and TIV as covariates. According to our hypothesis, we limited our search volumes to the brainstem within a mask created from the MNI152 atlas. A threshold for statistical significance was set at corrected *P* < 0.05 for multiple comparisons using threshold-free cluster enhancement (TFCE).

### *Ex vivo* MRI analysis

#### *Ex vivo* MRI data acquisition

To locate the brainstem nuclei of interest, we acquired *ex vivo* MRI data from 10 human brainstem specimens (one male and nine females; mean age, 91.0 ± 6.3 years; range, 77–97 years) that were free from known conditions affecting brainstem morphology (see Supplementary Table 3 for details). This study protocol was approved by the Kyoto University Ethics Committee (R3074).

All specimens underwent a standardized preparation protocol. After perfusion fixation and extraction, human brain specimens were maintained in 10% neutral buffered formalin at 4°C for at least 1 month, followed by more than four rounds of replacement with phosphate-buffered saline (PBS). Then the brainstem was dissected at the level of the mammillary bodies and immersed in PBS containing 10 mM gadoteridol for about 10 days to enhance the signal-to-noise ratio. Before scanning, each brainstem was fixed with sponges inside a plastic container filled with Fluorinert FC-43 (3M, Maplewood, MN, USA) and underwent a degassing procedure for half an hour to eliminate air bubbles that could affect image quality.

All *ex vivo* imaging was performed on a 7-Tesla MRI system for small animals (Bruker BioSpec 70/20 USR), equipped with a circular-polarized volume coil (72 mm inner diameter) for signal transmission and reception. The acquisition protocol employed a fast low-angle shot sequence (echo time/repetition time = 6.0/15.0 ms, flip angle = 60°) to achieve an isotropic resolution of 78.1 μm.

### Construction of probabilistic brainstem atlas

We applied a ‘prefiltered rotationally invariant non-local means filter’^30^ to enhance the signal-to-noise ratio of *ex vivo* MRIs while preserving fine anatomical details. Guided by the human brainstem atlas^31,32^ and the WIKIBrainStem template,^33,34^ we defined criteria for a set of brainstem nuclei. A trained neuroscientist outlined seven nuclei that are recognized components of REM sleep or arousal circuits: the PTg, the LDTg, the LDTgV, the LC and the SubC on each side, and the midline dorsal raphe nucleus (DR) and periaqueductal grey (PAG).^14,25,35-37^ We manually delineated volumes of interest (VOIs) on each sample’s MRI in its original high-resolution space. Another neuroscientist reviewed every contour, and disagreements were resolved by consensus. The comprehensive delineation protocol for each of the seven nuclei is provided in Supplementary Table 4. For instance, the PTg is located in the caudolateral midbrain tegmentum, extending from the midbrain–pontine junction, and is bordered ventrally by the superior cerebellar peduncle decussation. LDTgV is situated dorsolateral to the rostral pontine reticular formation, showing intermediate signal intensity, and bordered ventrally by the central tegmental tract, medially by the medial longitudinal fasciculus, dorsally by the LDTg and laterally by the LC. Most nuclei presented with relatively high signal intensities compared with the low signal intensities of adjacent white matter tracts. We were unable to delineate the SLD as a distinct VOI, primarily because its precise anatomical location in humans remains a subject of ongoing debate and is not consistently defined in existing human brain atlases.^31-34^ Instead, we defined the SubC and the LDTgV separately based on established criteria as candidate regions of the SLD homologue in humans.

The *ex vivo* MRI data exhibited inter-sample variability in size, shape and deformation, which resulted from the tissue fixation and imaging preparation processes. We developed and applied a dedicated image-processing pipeline to accommodate these variances and accurately transform each *ex vivo* MRI into the MNI152 space while minimizing variability. The spatial normalization of the *ex vivo* brainstem MRI data began with affine and non-linear diffeomorphic warping, referencing a brainstem tissue probability map adapted from the standard template. Optimal transformation parameters, determined by comparing results from standard normalization packages with Advanced Normalization Tools and SPM12, were applied to the VOIs derived from each sample’s original high-resolution space, yielding a 0.5-mm resolution probabilistic atlas in the MNI152 space. Finally, these spatially normalized VOIs were averaged across all samples, and voxels with a probabilistic distribution of 30% or greater for each nucleus were included in the present probabilistic brainstem atlas. The resulting 3D spatial relationship of these nuclei within the probabilistic atlas is illustrated in Fig. 1B.

**Figure 1:**
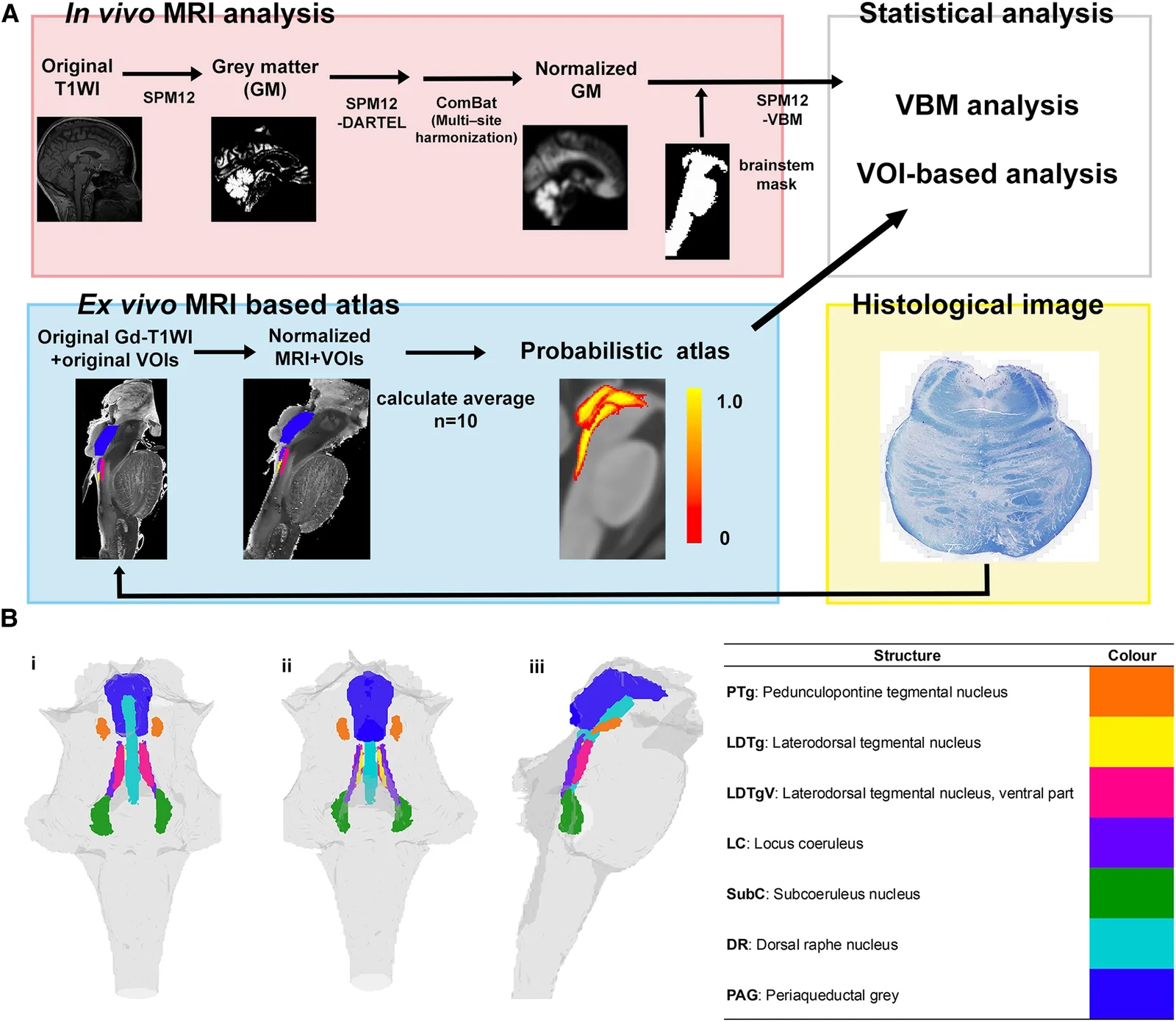
**Schematic of the study workflow and the resulting 3D probabilistic brainstem atlas.** (**A**) A schematic diagram of the study: (1) *in vivo* MRI analysis, including grey matter extraction, DARTEL normalization and ComBat harmonization; (2) *ex vivo* MRI-based probabilistic atlas construction from 10 post-mortem brainstems; (3) statistical analyses, including VBM and VOI analysis; and (4) histological validation. (**B**) Three-dimensional renderings of the probabilistic brainstem atlas. The spatial arrangement of the seven delineated brainstem nuclei is presented from (**i**) anterior, (**ii**) posterior and (**iii**) lateral perspectives. The colour key for each nucleus is provided on the right. Abbreviations: DARTEL, diffeomorphic anatomical registration through exponentiated Lie algebra; Gd, gadolinium; T1WI, T1-weighted imaging; VBM, voxel-based morphometry; VOI, volume of interest.

### Statistical analysis

#### Atlas-based analyses

We performed two complementary analyses using the probabilistic brainstem atlas to identify which nuclei might be affected in iRBD. First, we examined the spatial overlap between the GMV reduction identified in the VBM analysis and each nucleus defined in the atlas by calculating the proportion of each nucleus volume contained within the VBM cluster. Second, we extracted the effective GMV from each voxel within VOI by summing modulated GM values weighted by the atlas probabilities and multiplying by the voxel volume. The five bilaterally delineated VOIs (PTg, LDTg, LDTgV, LC and SubC) were averaged across the left and right sides to yield a single value per subject. The midline DR and PAG were each treated as a single VOI. Group comparisons were performed for the seven nuclei using a general linear model with age, sex and TIV as covariates. The family-wise error rate was controlled at an α of 0.05 using Bonferroni correction for multiple comparisons across the seven nuclei.

In addition to the bilaterally averaged analysis, we conducted supplementary analyses by examining each nucleus separately for each side. Group comparisons (iRBD versus HA) were performed using the same general linear model framework as the bilateral-pooled analysis (Bonferroni correction for 10 VOIs).

### Correlation with clinical measures

We investigated the correlation between clinical measures and the GMV of the brainstem nuclei that showed significant atrophy in iRBD patients. The primary clinical score of interest was the SCOPA-AUT total score, which assessed autonomic dysfunction in iRBD. As exploratory analyses, we also examined correlations with cognitive performance (MoCA-J) or motor impairment (MDS-UPDRS part III). Pearson’s partial correlation coefficients were calculated for these analyses, controlling for age, sex and TIV. Both GMV and clinical scores were residualized by regressing out the effects of these covariates, and the residuals were re-centred at the sample mean of each variable before plotting. All analyses were two-tailed. For all correlation analyses, the threshold was set at *P* < 0.05.

### Correlation with PSG measures

PSG data were not registered on the J-PPMI database. However, a subset of 23 iRBD patients still had detailed quantitative PSG data available, which were scored according to the American Academy of Sleep Medicine criteria. In these patients, we tested the hypothesis that the percentage ratio of REM sleep without atonia (RWA/REM%) was associated with GMV reduction of the affected nuclei in iRBD. We also examined the arousal index, periodic limb movement index (PLMI) and apnoea-hypopnoea index (AHI) as additional measures of sleep disturbance. Pearson’s partial correlation coefficients were computed with age, sex and TIV as covariates. GMV and PSG measures were residualized and re-centred before plotting, as described for the clinical measures. All analyses were two-tailed, with the threshold for significance set at uncorrected *P* < 0.05.

### Histological validation of the brainstem atlas

We performed histological validation of the brainstem nuclei that exhibited GMV reduction in iRBD. After the *ex vivo* MRI acquisition, the brainstem specimens were sectioned at a thickness of 15 μm. We performed Klüver–Barrera staining to visualize the general cyto/myeloarchitectural organization of the brain. For IHC, deparaffinized sections were subjected to antigen retrieval by autoclaving for 15 min, followed by membrane permeabilization with 0.2% Triton-X for 15 min. Endogenous peroxidase activity was quenched with 0.3% hydrogen peroxide for 30 min. After washing with PBS, sections were incubated with primary antibodies. We used a goat polyclonal anti-human choline acetyltransferase (ChAT) antibody [1:50; *E. coli*–derived recombinant human ChAT isoform R (Ala2–Pro630), AF3447, R&D Systems, Minneapolis, MN, USA] as a marker for cholinergic neurons and a rabbit polyclonal anti-human CRHBP antibody (1:100; human CRHBP amino acids 98–136, consistent with previous research^15^) as a marker for REM sleep-regulating neurons. The sections were treated with a secondary antibody (Histofine Simple Stain MAX-PO(G) for goat and MAX-PO(R) for rabbit; Nichirei Biosciences, Tokyo, Japan). The immunoreaction was visualized using 3,3′-diaminobenzidine with the VECTASTAIN ABC Kit (Vector Laboratories, Burlingame, CA, USA), followed by counterstaining with haematoxylin. Stained sections were imaged using an upright Axio Observer microscope (Carl Zeiss Microscopy GmbH, Germany) equipped with ZEN 3.8 software. The distribution patterns of CRHBP- and ChAT-immunoreactive neurons were compared with the VOIs on the *ex vivo* MRI.

To quantify the distribution of immunoreactive neurons, three representative post-mortem specimens of the 10 were analysed. The VOIs from our probabilistic brainstem atlas, corresponding to the anatomical levels of the midbrain, rostral pons and caudal pons, were overlaid onto the digitized histological images of the three representative specimens. Specifically, we used the PTg VOI at the midbrain level, the LDTgV at the rostral pontine level and the SubC at the mid-to-caudal pontine level. Within these atlas-defined regions, the number of ChAT- and CRHBP-positive cells was manually counted, and the area of each region was measured using QuPath v0.6.0.^38^ Cell density was then calculated and expressed as cells per square millimetre (cells/mm^2^).

## Results

### Demographic and clinical characteristics

The iRBD group [*n* = 98; mean age 70.8 ± 5.6 years; 73 males (74.5%)] and control HA group [*n* = 114; mean age 68.9 ± 5.7 years; 57 males (50.0%)] showed differences in age (*t*_(207)_ = −2.36, *P* = 0.019, two-tailed) and sex distribution (chi-square test, *P* < 0.001) (Table 1; see Supplementary Table 5 for characteristics by institution). Hence, age and sex were treated as confounding variables. The estimated MoCA-J scores^29^ of the control HA group (24.8 ± 2.7) showed no group-wise differences with the MoCA-J scores of the iRBD group (24.7 ± 3.1) (*t*_(193)_ = 0.41, *P* = 0.67, two-tailed).

**Table 1:** Demographic and clinical characteristics of iRBD and healthy aged control groups

	iRBD (*n* = 98)	HA (*n* = 114)	*P*-value
Age (years)	70.8 ± 5.6 (60–83)	68.9 ± 5.7 (60–83)	0.019*
Sex (male: female)	73: 25	57: 57	<0.001***
Institution	NCNP 54, Juntendo U. 20, Kyoto U. 12, Osaka U. 4, Nagoya U. 8	NCNP 83, Kyoto U. 31	
MoCA-J	24.7 ± 3.1 (16–30)	24.8 ± 2.7 (estimated)	0.67
MMSE	NA	28.7 ± 1.3 (24–30)	
SCOPA-AUT	9.7 ± 4.6 (2–24)	NA	
MDS-UPDRS Part III	2.6 ± 4.3 (0–31)	NA	
RBDSQ-J	7.4 ± 3.0 (0–12)	NA	

This table summarizes the baseline demographic and clinical characteristics of the iRBD and healthy aged control groups. Data are presented as mean ± standard deviation for continuous variables and as counts (male: female) for sex. Note that the MoCA-J scores for the healthy aged group were estimated from Mini-Mental State Examination scores.

A breakdown of demographic and clinical characteristics for each recruitment institution is provided in Supplementary Table 5. *P < 0.05; ***P < 0.001.

### VBM analysis

The VBM analysis identified a single cluster of GMV reduction in patients with iRBD compared with HAs. This cluster was located in the midbrain and upper pontine tegmentum (peak MNI coordinate: *x* = −4, *y* = −34, *z* = −21; peak *t* = 4.01; 469 voxels; TFCE-corrected *P* < 0.05; Fig. 2). Our probabilistic brainstem atlas indicated that this cluster included the bilateral LDTgV, PTg, LDTg and SubC. A semi-quantitative assessment of spatial overlap revealed that 70.6% of the LDTgV volume was contained within the VBM cluster, representing the highest degree of overlap among all examined nuclei, followed by the PTg (45.9%) and LDTg (25.0%) (see Supplementary Fig. 2 for details).

**Figure 2:**
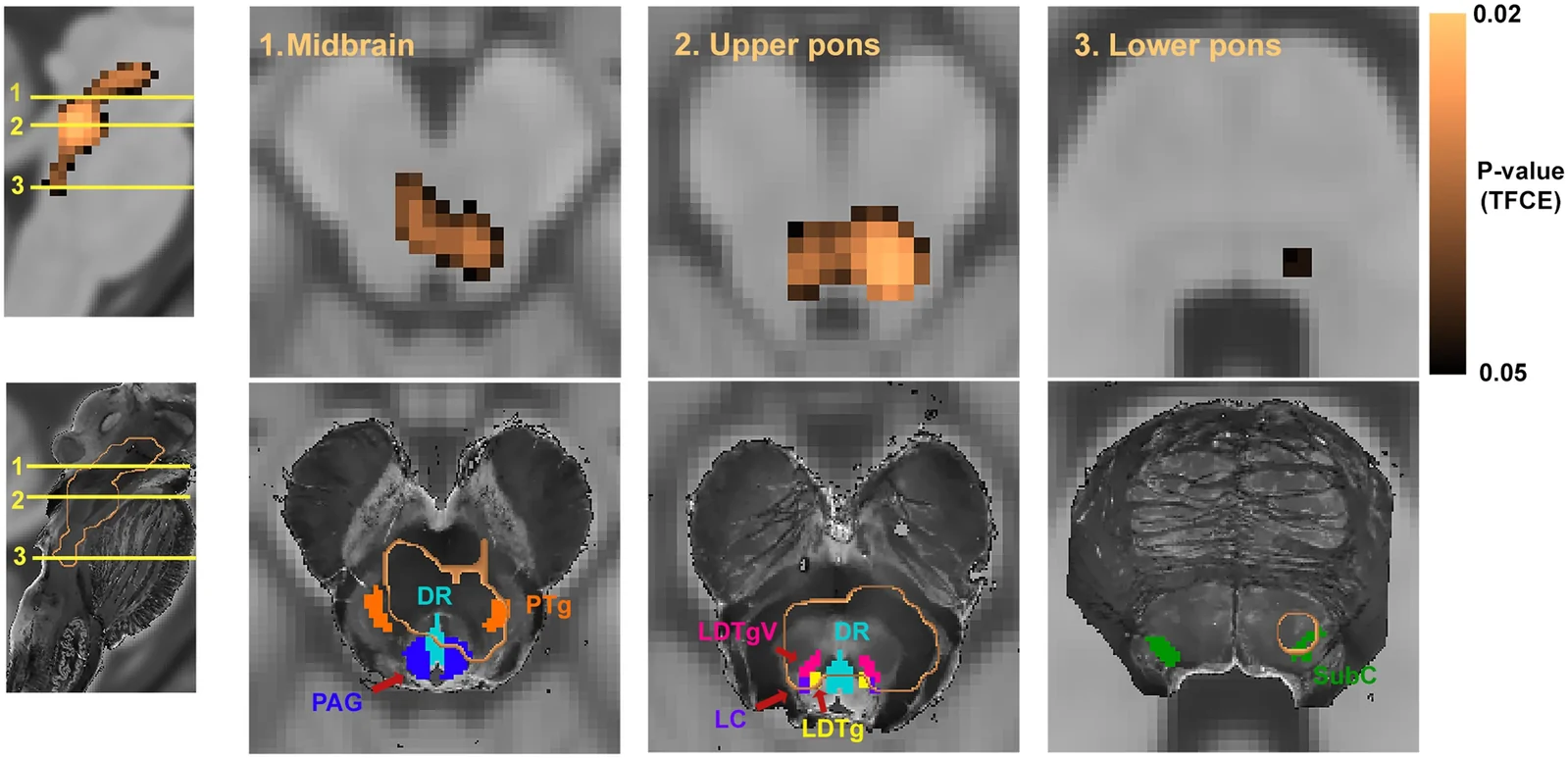
**GMV reduction in iRBD.** Results of the voxel-based morphometry analysis (iRBD, *n* = 98; HA, *n* = 114). The top row displays a cluster of significant GMV reduction (copper) in the midbrain and upper pontine tegmentum in iRBD compared with controls, identified using a general linear model with age, sex and total intracranial volume as covariates and assessed by TFCE, *P* < 0.05 corrected (peak *t* = 4.01, 469 voxels). The bottom row shows the VBM results (copper outlined) overlaid on the *ex vivo* brainstem atlas, demonstrating spatial overlap of the atrophy with the LDTgV, PTg, LDTg and SubC. The colour bar indicates the *P*-value. Abbreviations: GMV, grey matter volume; HA, healthy aged controls; iRBD, isolated REM sleep behaviour disorder; LDTg, laterodorsal tegmental nucleus; LDTgV, laterodorsal tegmental nucleus, ventral part; PTg, pedunculopontine tegmental nucleus; SubC, subcoeruleus nucleus; TFCE, threshold-free cluster enhancement; VBM, voxel-based morphometry.

### Atlas-based analysis

In the atlas-based analysis of GMV in the seven brainstem nuclei, we found significant GMV reductions in the LDTgV of patients with iRBD compared with HAs [mean difference = 0.58, 95% confidence interval (CI) (0.20, 0.95), Bonferroni-corrected *P* = 0.019, Cohen’s *d* = 0.44; Fig. 3], after controlling for confounding variables. Similarly, the PTg showed reduced GMV in iRBD patients compared with HAs [mean difference = 0.33, 95% CI (0.09, 0.57), Bonferroni-corrected *P* = 0.049, Cohen’s *d* = 0.40; Fig. 3]. By contrast, GMV in the other five nuclei—the LDTg, LC, SubC, DR and PAG—did not differ significantly between the iRBD and HA groups (Bonferroni-corrected *P* > 0.05).

**Figure 3:**
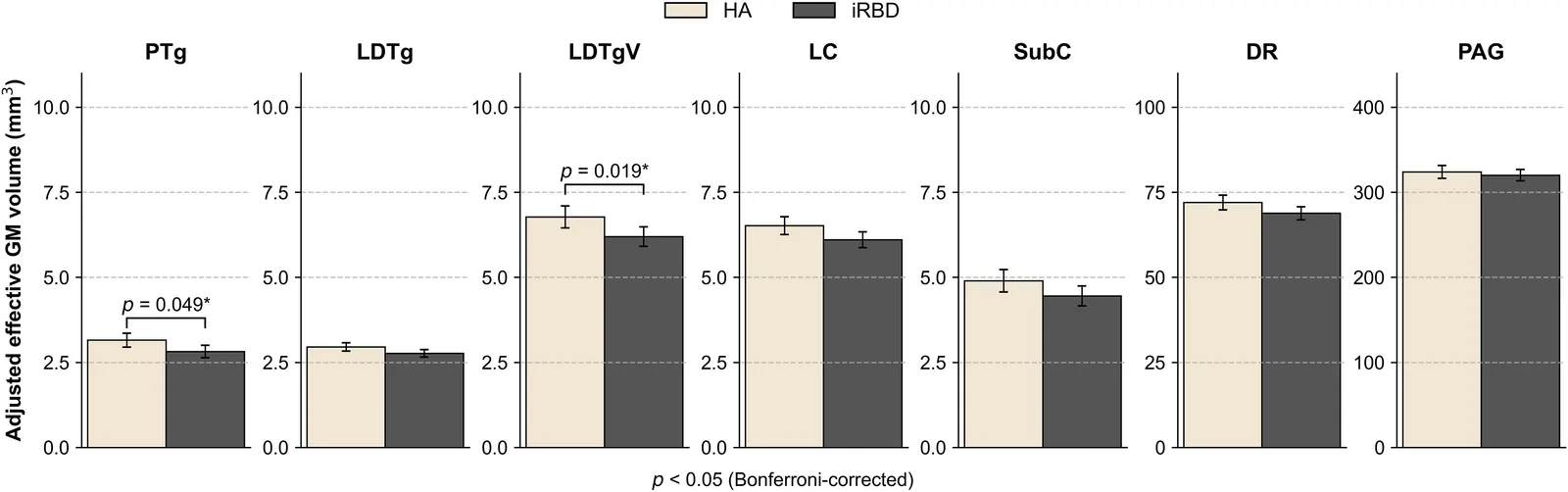
**VOI-based grey matter volume analysis of brainstem nuclei.** The comparison of adjusted grey matter volume in the seven brainstem nuclei between iRBD (*n* = 98, dark bars) and HA controls (*n* = 114, light bars). Group comparisons used a general linear model with age, sex and total intracranial volume as covariates, with Bonferroni correction across the seven nuclei. After correction, significant volume reduction was identified in the LDTgV (mean difference = 0.58, Cohen’s *d* = 0.44, Bonferroni-corrected *P* = 0.019) and the PTg (mean difference = 0.33, Cohen’s *d* = 0.40, Bonferroni-corrected *P* = 0.049). Error bars represent 95% confidence intervals. The *y*-axis shows adjusted grey matter volume. Abbreviations: HA, healthy aged controls; iRBD, isolated REM sleep behaviour disorder; LDTg, laterodorsal tegmental nucleus; LDTgV, laterodorsal tegmental nucleus, ventral part; LC, locus coeruleus; PTg, pedunculopontine tegmental nucleus; SubC, subcoeruleus nucleus; DR, dorsal raphe nucleus; PAG, periaqueductal grey; VOI, volume of interest.

A supplementary analysis examining the left and right nucleus separately (Bonferroni-corrected) supported significant atrophy of the LDTgV on both sides and of the PTg on the left side (Supplementary Table 6).

### Correlation analysis with clinical measures

We investigated the correlation between clinical scores and atrophy in the LDTgV and the PTg, which showed structural alterations in iRBD. We focused on the correlates of autonomic dysfunction, as measured by SCOPA-AUT, after controlling for age, sex and TIV. Partial correlation analysis revealed that smaller GMV in the LDTgV and the PTg was associated with higher standardized SCOPA-AUT scores [LDTgV: partial *r* = −0.237; 95% CI (−0.418, −0.037), *P* = 0.021, Fig. 4A; PTg: partial *r* = −0.236; 95% CI (−0.418, −0.037), *P* = 0.021, Fig. 4B], suggesting that greater atrophy in these pontine tegmental nuclei was associated with more severe autonomic dysfunction in patients with iRBD. We also conducted exploratory correlation analyses to investigate the relationship between the volumes of the LDTgV and PTg and other clinical domains, including cognitive function (MoCA-J) and motor impairment (MDS-UPDRS-III). None of these scores yielded statistically significant correlations with the LDTgV and PTg (all *P* > 0.05, uncorrected).

**Figure 4:**
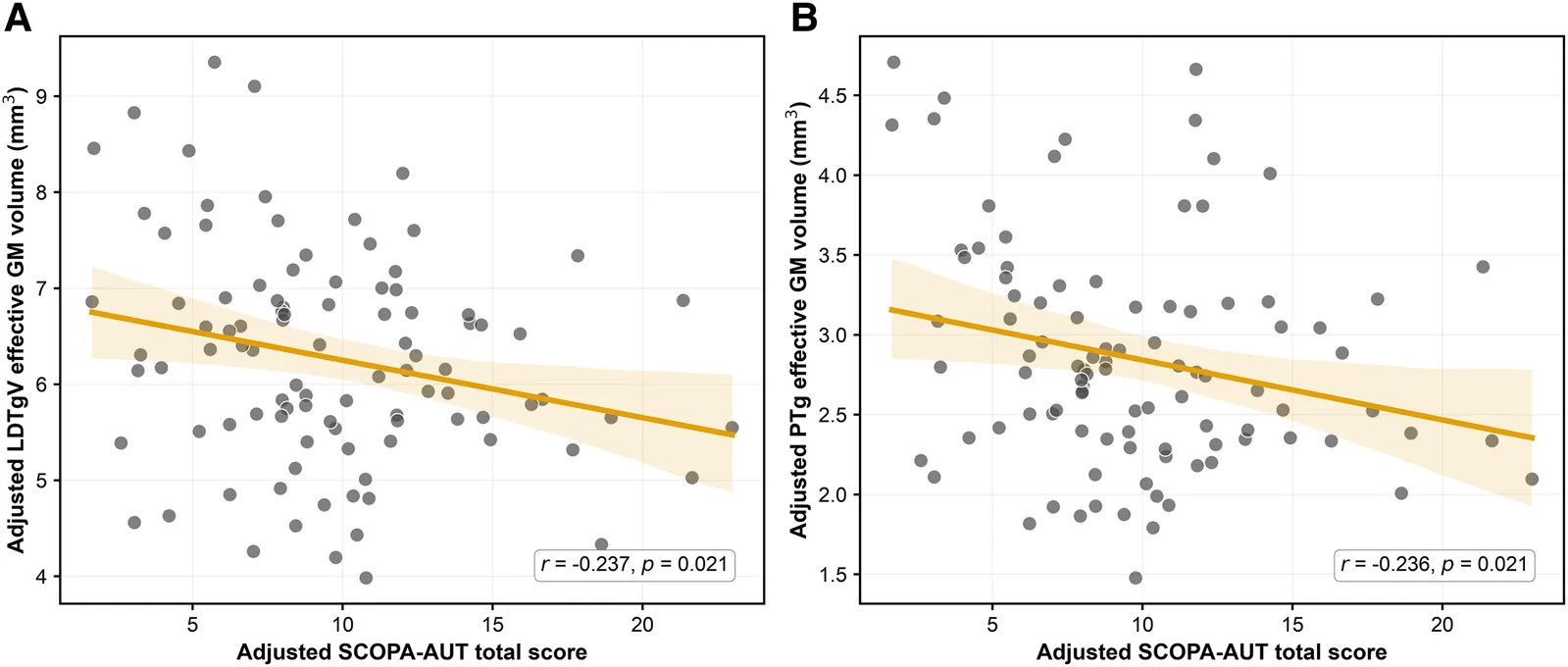
**Correlation between brainstem nuclei volume and autonomic dysfunction.** Scatter plots of the relationship between adjusted GMV and the SCOPA-AUT scores in patients with iRBD (*n* = 98). Associations were tested with Pearson’s partial correlation analysis controlling for age, sex and TIV. (**A**) A significant negative correlation between LDTgV volume and SCOPA-AUT score [partial *r* = −0.237, 95% CI (−0.418, −0.037), *P* = 0.021]. (**B**) A significant negative correlation between PTg volume and SCOPA-AUT score [partial *r* = −0.236, 95% CI (−0.418, −0.037), *P* = 0.021]. Solid lines represent the line of best fit; shaded areas represent the 95% confidence intervals. Abbreviations: iRBD, isolated REM sleep behaviour disorder; LDTgV, laterodorsal tegmental nucleus, ventral part; PTg, pedunculopontine tegmental nucleus; SCOPA-AUT, Scales for Outcomes in Parkinson's Disease-Autonomic Dysfunction.

### Correlation analysis with PSG measures

Partial correlation analysis in the 23 iRBD patients with PSG data revealed that atrophy of the LDTgV was associated with higher RWA/REM% [partial *r* = −0.491, 95% CI (−0.767, −0.062), *P* = 0.028, Fig. 5A]. A non-significant association in the same direction was observed for the PTg [partial *r* = −0.379, 95% CI (−0.703, 0.077), *P* = 0.100, Fig. 5B]. Neither nucleus showed significant correlation with the arousal index, PLMI or AHI (all *P* > 0.05, uncorrected; Supplementary Table 7), indicating that the LDTgV atrophy was specifically related to REM atonia.

**Figure 5:**
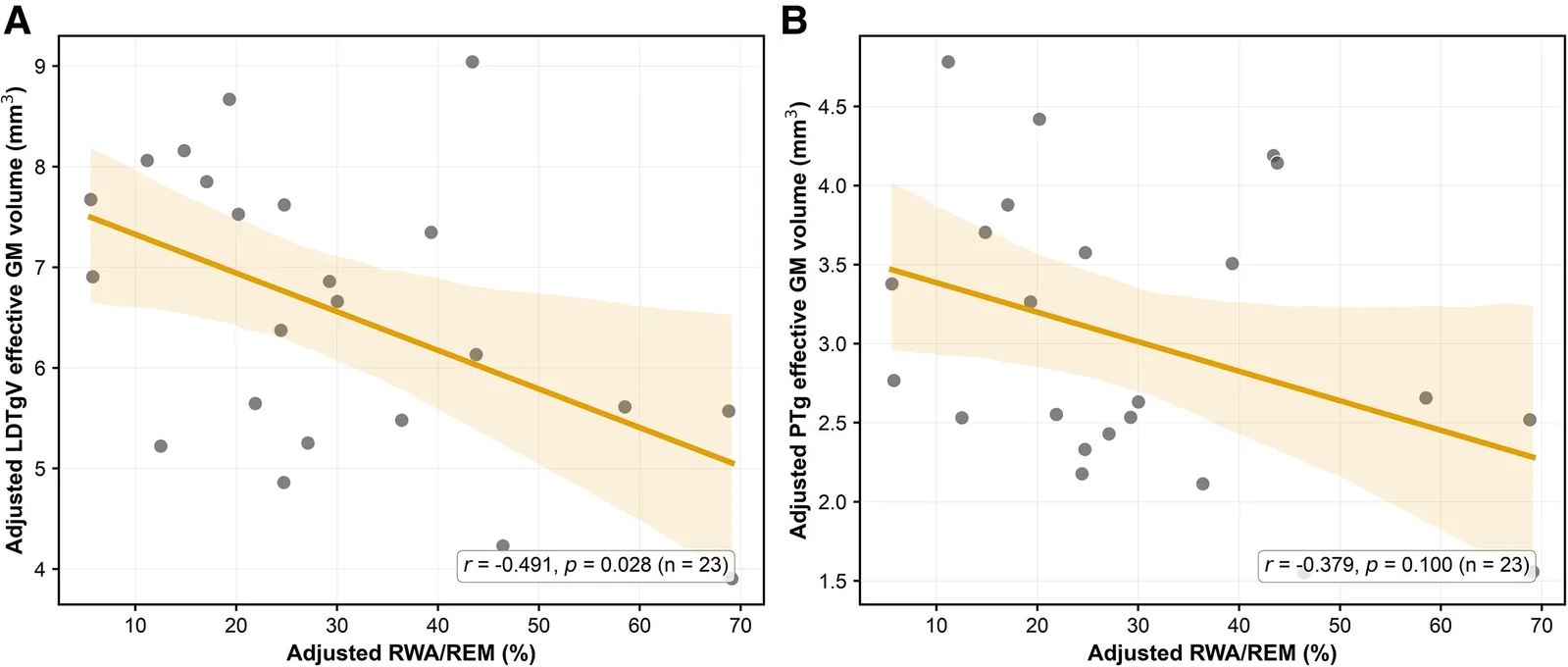
**Correlation between brainstem nuclei volume and REM sleep without atonia.** Scatter plots of the relationship between adjusted GMV and the percentage ratio of REM sleep without atonia (RWA/REM%) in patients with iRBD with quantitative polysomnography (*n* = 23). Associations were tested with Pearson’s partial correlation analysis controlling for age, sex and TIV. (**A**) A significant negative correlation between LDTgV volume and RWA/REM% [partial *r* = −0.491, 95% CI (−0.767, −0.062), *P* = 0.028]. (**B**) A non-significant association in the same direction for the PTg [partial *r* = −0.379, 95% CI (−0.703, 0.077), *P* = 0.100]. Solid lines represent the line of best fit; shaded areas represent the 95% confidence intervals. Each point represents one of the 23 iRBD patients. Abbreviations: iRBD, isolated REM sleep behaviour disorder; LDTgV, laterodorsal tegmental nucleus, ventral part; PTg, pedunculopontine tegmental nucleus; RWA, REM sleep without atonia.

### Histological validation

Using post-mortem human brainstem specimens, we conducted histological examinations of LDTgV, PTg, LDTg, LC, SubC and DR. The Klüver–Barrera staining delineated the general cyto- and myeloarchitecture. It supported that the cellular arrangements and fibre tract patterns (Fig. 6, Klüver–Barrera columns) corresponded well with the anatomical segmentations of the DR, PTg, LDTg, LDTgV, LC and SubC between the *ex vivo* MRI and histology (Fig. 6A left columns). Consistent with established neuroanatomy, ChAT-positive cholinergic neurons were observed within the LDTg and LDTgV (Fig. 6A middle row, ChAT column), as well as in the PTg (Fig. 6A top row, ChAT column). IHC for CRHBP revealed that CRHBP-immunoreactive neurons were predominantly distributed within the LDTgV (Fig. 6A middle row, CRHBP column, arrowheads; lower row). By contrast, in the adjacent LDTg and other examined nuclei, including the DR, PTg, LC and SubC, CRHBP-immunoreactive neurons were relatively sparse or absent under the identical IHC condition. This specific pattern of CRHBP immunoreactivity within the LDTgV, when considered alongside the significant atrophy of the LDTgV observed in iRBD *in vivo*, suggests that this neuronal population is preferentially targeted by the underlying pathological process. Quantitative analysis of the three representative specimens supported these observations, revealing distinct distribution patterns of CRHBP- and ChAT-immunoreactive neurons across the brainstem nuclei (Fig. 6B). The density of CRHBP-positive neurons was highest in the LDTgV (12.08 ± 2.64 cells/mm^2^), followed by the PTg (3.73 ± 1.31 cells/mm^2^), and was minimal in the SubC (0.96 ± 0.66 cells/mm^2^). ChAT-positive neurons were most densely distributed in the PTg (30.26 ± 14.53 cells/mm^2^) and LDTgV (19.57 ± 7.29 cells/mm^2^), with the lowest density in the SubC (2.06 ± 1.14 cells/mm^2^). This quantitative data reinforces that CRHBP-expressing neurons, implicated in regulating REM sleep, are particularly concentrated within the LDTgV, the putative human homologue of the murine SLD, located in the rostral pontine tegmentum.

**Figure 6:**
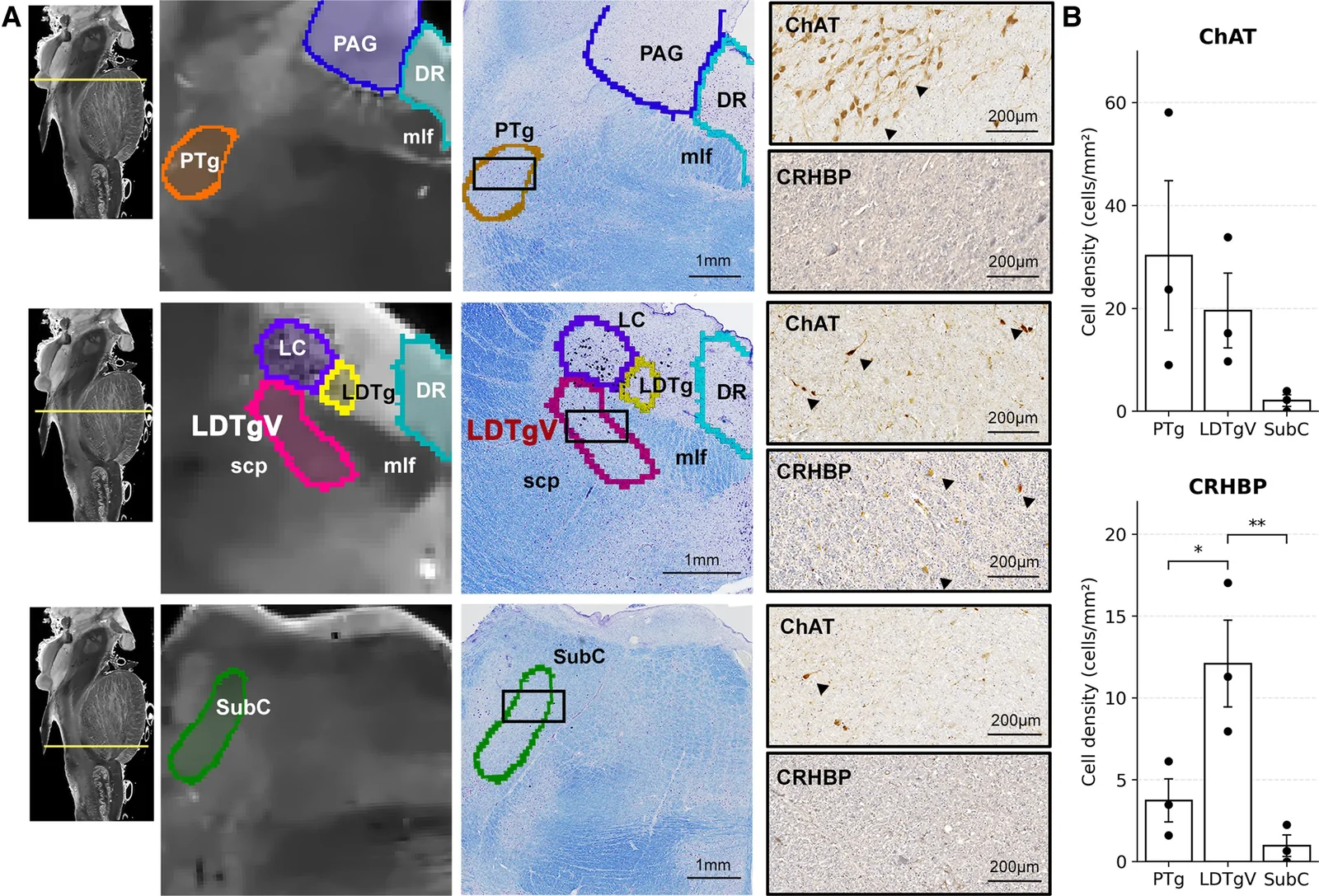
**Histological validation of brainstem nuclei.** (**A**) Each row corresponds to a brainstem level shown in the sagittal MRI. Columns from left to right show: (1) *ex vivo* MRI with colour-coded nuclei delineations; (2) Klüver–Barrera staining for cyto- and myeloarchitecture; (3) ChAT immunohistochemistry (corresponding to the black rectangle in 2); (4) CRHBP immunohistochemistry. The areas in 3 and 4 correspond to the black rectangle in 2. Arrowheads indicate immunoreactive neurons. CRHBP-positive neurons, a marker for REM sleep-on neurons, were localized to the LDTgV. (**B**) Quantitative cell density analysis of ChAT- and CRHBP-immunoreactive neurons in the PTg, LDTgV and SubC. Cell densities were calculated from three post-mortem human specimens (*n* = 3 brains). The density of CRHBP-positive neurons was markedly higher in the LDTgV compared with the other nuclei. Values are presented as mean ± standard error of the mean (SEM). Statistical comparisons were made using a one-way ANOVA followed by Tukey’s *post hoc* test. For CRHBP, significant differences were found between PTg and LDTgV (* *P* < 0.05) and between LDTgV and SubC (** *P* < 0.01). ChAT-positive neurons were distributed across all three nuclei without significant differences. Abbreviations: LDTgV, laterodorsal tegmental nucleus, ventral part; PTg, pedunculopontine tegmental nucleus; scp, superior cerebellar peduncle; SubC, subcoeruleus nucleus.

## Discussion

We analysed structural MRI data from a cohort of iRBD and HAs, guided by a newly developed histology-validated human brainstem atlas based on *ex vivo* 7-T MRI. The VBM findings showed GMV reductions in the midbrain and upper pontine tegmentum in iRBD. The atlas-based volumetric analysis revealed atrophy in both the LDTgV and the PTg in iRBD compared with HAs, with the LDTgV exhibiting the most pronounced GMV reduction among the brainstem nuclei examined. The supplementary analysis on each side supported LDTgV atrophy on both sides and PTg atrophy on the left side. The right PTg also showed a tendency. Earlier structural MRI studies have implicated brainstem abnormalities in iRBD, but the involved nuclei have remained inconsistent across reports. For instance, some VBM reports indicated GMV reductions in the pontine tegmentum,^39^ while others showed more diffuse GMV loss in the brainstem^40^ or, conversely, no significant GMV changes in the brainstem.^41^ Indeed, a meta-analysis of VBM literature has not identified a uniform pattern of brainstem atrophy in iRBD,^19^ reflecting not only methodological differences across studies but also challenges in resolving small brainstem nuclei with standard technology. Other imaging modalities have also indicated brainstem tegmental abnormalities in iRBD. Diffusion MRI has shown reduced fractional anisotropy in the midbrain tegmentum and rostral pons, with increased mean diffusivity in the pontine reticular formation,^41^ while another study reported reduced axial diffusivity in the substantia nigra and pons.^42^ A study using an *in vivo* probabilistic brainstem atlas and diffusion tractography reported altered pontomedullary connectivity in iRBD.^43^ Neuromelanin-sensitive MRI has shown reduced signal in the locus coeruleus/subcoeruleus complex in iRBD,^36^ and brain perfusion measured during RBD episodes has revealed activation in the dorsal and ventral pontine tegmentum.^44^ The heterogeneity of these earlier reports may reflect both methodological differences and the difficulty of resolving small adjacent brainstem nuclei with conventional *in vivo* imaging. The brainstem tegmentum contains many small nuclei and different fibre tracts. The exact boundaries of nuclei remain debated.^45^ Recent histological and ultra-high-field *ex vivo* MRI atlases of the human brainstem provide essential anatomical references for defining these structures.^31-34^ Building on these previous resources, we created novel probabilistic VOIs that could be directly overlaid onto *in vivo* MRI results, allowing us to interpret tegmental atrophy at the level of the LDTgV and PTg rather than broadly to the pontine tegmentum. Thus, by relating the VBM findings to candidate nuclei, our findings provide a more anatomically precise account of brainstem involvement in iRBD.

Our IHC analysis revealed that the LDTgV harbours CRHBP-expressing neurons, a neuronal population known to regulate REM sleep and the associated muscle atonia. The human homologue of the murine SLD has been a subject of debate. Previous literature often regarded the SubC as this human equivalent^25^; however, the supporting evidence remains limited. In fact, this small REM-regulating tegmental region has been variably termed the peri-locus coeruleus alpha in cats and either the sublaterodorsal/subLDT nucleus or the ventral part of the laterodorsal tegmental nucleus (LDTgV) in rodents, with substantial inconsistency across species, atlases and investigators.^14,25,26^ Another line of evidence, based on detailed studies of the rat ponto-mesencephalic tegmentum, suggests that the LDTgV may correspond to the murine SLD.^46,47^ A recent electrophysiological study in mice has referred to the subLDT as corresponding to the LDTgV,^48^ providing a converging anatomical correspondence across rodent species. Such anatomical differentiation in humans has remained difficult with conventional *in vivo* MRI or routine histology, given the close proximity of the SubC, LDTgV and adjacent nuclei, and most prior studies have treated the SubC as part of the locus coeruleus/subcoeruleus complex.^21^ Our histology-validated *ex vivo* 7-T atlas delineates the SubC and the LDTgV as distinct entities, and applying this atlas to *in vivo* MRI together with CRHBP immunohistochemistry on post-mortem specimens allowed direct comparison of the two candidate nuclei within the same anatomical framework. CRHBP immunoreactivity was predominantly localized to the LDTgV, whereas the SubC showed only sparse CRHBP immunoreactivity. The LDTgV showed pronounced atrophy on *in vivo* MRI in patients with iRBD, whereas the SubC did not. Therefore, our anatomical and imaging findings favour a view that the LDTgV, rather than the SubC, is a candidate human counterpart of the murine SLD.

Despite the results from a limited subset, we found that smaller LDTgV volume was associated with longer REM sleep without atonia. The PTg showed a similar trend. This finding, in part, aligns with prior reports of negative correlations between neuromelanin signal intensity in the locus coeruleus/subcoeruleus complex and RWA/REM% in iRBD^36^ and in Parkinson’s disease with RBD.^21^ These earlier studies and ours commonly identified a similar dorsolateral pontine region underlying iRBD. Although our multimodal data converged to identify the LDTgV, the question of whether the LDTgV or the locus coeruleus/subcoeruleus complex is the critical RWA centre needs to be solved in larger longitudinal cohorts.

The CRHBP-expressing neurons within the murine SubLDT/SLD have been shown to have an important role in REM sleep atonia.^15^ In our study, CRHBP-immunoreactive neurons were concentrated in the atlas-defined human LDTgV, the same nucleus that showed the most consistent atrophy in iRBD. These findings link the animal evidence for CRHBP-positive REM-atonia neurons to a human pontine tegmental nucleus affected in iRBD. The rodent SubLDT/SLD provides a major pontine excitatory input to GABAergic and glycinergic premotor neurons of the ventromedial medulla, which inhibit spinal motoneurons.^49,50^ For instance, targeted ablation of these *Crhbp*-positive SLD neurons markedly reduces REM sleep and impairs REM atonia in mice,^15^ and single-unit recordings have shown that medulla-projecting *Crhbp*-positive neurons are predominantly REM sleep-active, with firing rates that correlate negatively with EMG activity during REM sleep.^48^ The PTg, another REM-related nucleus that showed atrophy in our iRBD cohort, showed only sparse CRHBP immunoreactivity, further highlighting the unique neurochemical profile of the LDTgV. Our current findings, combined with the animal evidence for the role of CRHBP neurons in REM atonia, suggest that the human LDTgV is a critical node of the REM-atonia circuit affected in iRBD.

Beyond its role in REM atonia, the LDT complex serves as an integrator of brainstem functions characterized by extensive connectivity. This aligns with evidence from tract-tracing studies, which show projections from the LDT complex to the thalamus, hypothalamus, basal forebrain and frontal cortex, as well as to other brainstem nuclei, including the PTg.^51^ The LDT complex is implicated not only in REM sleep and arousal^52^ but also in autonomic regulation via the nucleus of the solitary tract and parabrachial nuclei.^51^ Hence, the atrophy of the LDTgV likely extends its functional impact to a broader array of non-motor symptoms, including autonomic dysfunctions, which are frequently associated with iRBD. This notion is supported by a significant association between LDTgV atrophy and SCOPA-AUT scores, which aligns with a previous large-scale network study.^40^ The LDTgV, with its established connections to autonomic centres and its demonstrated atrophy in iRBD, thus represents an anatomical substrate related to both REM sleep dysregulation and coexisting autonomic disturbances. Identifying such specific brainstem correlates of non-motor symptoms in iRBD was made possible by the anatomical precision afforded by our high-resolution, histologically validated atlas, which delineates the LDTgV from adjacent structures and quantifies these structural changes.

The LDTgV atrophy observed in our cohort may represent a brainstem structural correlate of REM-atonia dysfunction within the broader neurodegenerative process of iRBD. A previous post-mortem study reported α-synuclein pathology in CRHBP-expressing neurons within the putative human SubLDT region of the pontine tegmentum in patients with Parkinson’s disease and RBD, corresponding to the atlas-defined LDTgV.^15^ A recent pathological study on individuals with pre-mortem diagnosis of iRBD further showed α-synuclein pathology and neuronal loss in REM-atonia-related structures, including the subcoeruleus nucleus, gigantocellular reticular nucleus, laterodorsal tegmentum, lateral pontine tegmentum–pedunculopontine nucleus and amygdala.^53^ These observations demonstrated the involvement of multiple components of the REM-atonia network. This study also showed frequent Alzheimer-type and other age-related concomitant pathologies, indicating that the pathological background of iRBD is heterogeneous even though α-synuclein pathology is central.^53^ Dopaminergic PET and dopamine-transporter SPECT studies have also shown early nigrostriatal involvement in idiopathic RBD, including reduced vesicular monoamine transporter binding and dopamine-transporter uptake.^2,54-56^ The post-mortem and dopaminergic imaging literature suggests that, by the clinical stage of iRBD, pathological changes may already extend beyond the pontine tegmentum to wider REM-atonia-related, limbic and nigrostriatal systems.

Our study had several limitations. First, this was a cross-sectional study, and longitudinal data analysis will be desirable to track LDTgV and PTg atrophy and to assess their predictive value for conversion to α-synucleinopathies. Second, the PSG correlation analysis was performed only in 23 patients out of 98, requiring replication in larger independent cohorts. Third, we did not have neuropathological data from the patients with iRBD. The correspondence between the observed atrophy and α-synuclein pathology can therefore be supported only by previous post-mortem reports, and future imaging–pathology studies will be needed to confirm it directly. Fourth, the absence of significant atrophy in atlas-defined nuclei other than the LDTgV and PTg should not be interpreted as evidence against their involvement in RBD. T1-weighted VBM may not capture all relevant pathological changes, including α-synuclein pathology, gliosis, neurotransmitter-specific neuronal loss or microstructural alterations. Fifth, our analysis focused on the midbrain and pontine nuclei because the VBM cluster was localized there. We thus concentrated on delineating REM-related and arousal-related nuclei at these levels. Consequently, medullary structures such as the gigantocellular reticular nucleus and adjacent ventromedial medulla, which house GABAergic and glycinergic premotor neurons critical for REM atonia,^49,50^ were not included in our analysis. Hence, the medullary structure was outside of this study’s scope. Sixth, the PSG or RBDSQ-J was unavailable from HA. Therefore, unrecognized RBD or subclinical REM sleep without atonia cannot be completely excluded. Given the low prevalence of RBD in the general population, the number of individuals with RBD is likely to be small. Nevertheless, the possibility that undetected cases reduced the apparent group differences cannot be excluded, and this should be considered when interpreting nuclei that did not show statistical differences. Finally, regarding the *ex vivo* atlas itself, tissue fixation introduced anatomical variability that may affect boundary precision between adjacent nuclei. The probabilistic nature of our atlas, integrating 10 specimens, partly accounts for inter-individual variation but cannot fully remove uncertainty in small-nucleus boundaries. Future development of automated segmentation methods using machine learning approaches may further improve the objectivity and reproducibility of brainstem nuclei delineation.

In conclusion, we provide robust evidence that iRBD is associated with atrophy of the LDTgV, a CRHBP-rich pontine tegmental nucleus, that we considered a human homologue of the murine SLD. This degeneration of the LDTgV, alongside the PTg, was associated with autonomic dysfunction and with the severity of REM sleep without atonia, supporting a contributory role of these pontine tegmental structures in the pathophysiology of iRBD. These findings enhance our understanding of the neuroanatomical basis of iRBD and highlight the LDTgV as a potential anatomical site for developing a biomarker for prodromal α-synucleinopathy.

## Supplementary Material

fcag379_Supplementary_Data

## Data Availability

The probabilistic brainstem atlas generated in this study is available in the Kyoto University Research Information Repository (KURENAI) under a Creative Commons Attribution-NonCommercial-NoDerivatives 4.0 International (CC BY-NC-ND 4.0) licence at http://hdl.handle.net/2433/301268 (DOI: https://doi.org/10.57723/kds622523). The raw *ex vivo* magnetic resonance imaging data and full-resolution histological images are available from the corresponding author upon reasonable request. The de-identified individual-level *in vivo* magnetic resonance imaging and clinical data from the J-PPMI cohort are not publicly available due to privacy regulations and stipulations within the participant consent forms. All analyses used standard, publicly available software. The analysis scripts are available from the corresponding author upon reasonable request.
