# Supplementary material for: Tegmental atrophy in isolated REM sleep behaviour disorder: ex vivo MRI–informed in vivo imaging
Source: Brain Commun. 2026 Oct 8;8(5):fcag379. doi: 10.1093/braincomms/fcag379 (PMC13691600; doi:10.1093/braincomms/fcag379)
Supplement: Supplementary file 1 [file fcag379_supplementary_data.pdf]

Supplementary Table 1: MRI acquisition parameters at each institution.

Sites	Scanner	Sequence	TR (ms)	TE (ms)	TI (ms)	FA (degree)	Resolution (mm)
NCNP	Siemens Verio Dot	MPRAGE	1900	2.5	900	9	1.0×1.0×1.0
Juntendo	Philips Achieva	MPRAGE	15	3.4	1022	10	1.0×1.0×1.0
Kyoto	Siemens Skyra	MPRAGE	1900	2.6	900	9	0.9×0.9×0.9
Osaka	GE Discovery MR 750	IR-SPGR	8.2	3.2	400	11	1.2×1.2×1.2
Nagoya	Siemens Verio	MPRAGE	2500	2.5	900	8	1.0×1.0×1.0

Detailed imaging parameters for the 3-dimensional T1-weighted imaging (3DT1WI) sequences used at each site. Information listed includes scanner manufacturer, sequence name, repetition time (TR), echo time (TE), inversion time (TI), flip angle (FA), and resolution.

Abbreviations: NCNP, National Center of Neurology and Psychiatry; Juntendo Univ., Juntendo University; Kyoto Univ., Kyoto University; Osaka Univ., University of Osaka; Nagoya Univ., Nagoya University.

Supplementary Table 2: Assessment of site effect reduction by ComBat harmonization

VOI	F_Before	F_After	η^2_Before	η^2_After
**PTg**	21.29	2.01	0.30	0.04
**LDTg**	11.38	0.90	0.18	0.02
**LDTgV**	13.86	1.14	0.21	0.02
**LC**	10.62	0.92	0.17	0.02
**SubC**	9.41	1.15	0.16	0.02
**PAG**	25.09	2.15	0.33	0.04
**DR**	21.34	1.72	0.30	0.03

This table provides quantitative metrics assessing the magnitude of the site effect on grey matter volume for each brainstem nucleus before and after ComBat harmonization. The magnitude of the site effect is reported using the F-statistic (F) and partial eta-squared (η^2) for the site term in an ANCOVA with age, sex, TIV and

diagnostic group as covariates. ComBat reduced the site effects in all nuclei. Data from the left and right sides were averaged, except for the midline structures (PAG and DR). Abbreviations: Pedunculopontine tegmental nucleus (PTg), Laterodorsal tegmental nucleus (LDTg), Laterodorsal tegmental nucleus, ventral part (LDTgV), Locus coeruleus (LC), Subcoeruleus nucleus (SubC), Dorsal raphe nucleus (DR), Periaqueductal grey (PAG)

Supplementary Table 3: Details of human brainstem specimens used for *ex vivo* MRI.

Case	Age of death	Sex	Cause of death
1	94	Female	Chronic kidney disease
2	97	Female	Aspiration pneumonia
3	87	Male	Aspiration pneumonia
4	77	Female	Ischemic heart disease
5	89	Female	Sepsis
6	97	Female	Senility
7	97	Female	Senility
8	87	Female	Strangulated ileus
9	91	Female	Acute myocardial infarction
10	94	Female	Cardioembolic stroke

This table provides the demographic and specimen characteristics for the 10 *postmortem* human brainstem samples used for the high-resolution *ex vivo* MRI analysis.

Supplementary Table 4: Delineation Criteria for Brainstem Nuclei in *Ex vivo* MRI Atlas.

Structure	Anatomical Location and MRI Characteristics
PTg (Pedunculopontine tegmental nucleus)	Located in the reticular region of the caudolateral midbrain tegmentum, extending from the midbrain-pontine junction. Bordered dorsally by the isthmic reticular formation and ventrally by the superior cerebellar peduncle decussation.
LDTg (Laterodorsal tegmental nucleus)	Situated laterally in the pontine central grey, extending from the midbrain-pontine boundary to the mid-pons. Presents as a mildly hyperintense structure adjacent to LC. Bordered by the mlf and LDTgV ventrally, surrounded by the central grey matter.
LDTgV (Laterodorsal tegmental nucleus, ventral part)	Positioned dorsolateral to the rostral pontine reticular formation. Shows intermediate signal intensity (higher than the surrounding pontine reticular region, lower than the central grey). Bordered by the PnO and the ctg ventrally, the mlf medially, the LDTg dorsally, and the LC laterally
LC (Locus coeruleus)	Located laterally in the pontine tegmentum throughout the pons. Identified by characteristic "pepper-and-salt" appearance due to neuromelanin. Appears as a round structure axially. Surrounded by PnO, LDTgV, LDT, and superior cerebellar peduncle.
SubC (Subcoeruleus nucleus)	Presents as a faint hyperintense area in the lateral lower pons, caudal to LC. Extends ventrally in pontine reticular formation around Mo5N medially, terminating at the rostral end of the facial nucleus (7N).
DR (Dorsal raphe nucleus)	Located ventral to the cerebral aqueduct, appearing as an elongated hyperintense structure extending axially. Bounded ventrolaterally by the mlf. The lateral boundary with PAG is defined by three lines extending to the cerebral aqueduct: caudally, a line from the dorsolateral angle of the mlf to the midline of the cerebral aqueduct; at mid-level, a line from the lateral margin of the trochlear nucleus (4N); and rostrally, a line from the lateral margin of the oculomotor nucleus (3N)
PAG (Periaqueductal grey)	High-signal grey matter structure surrounding the cerebral aqueduct in the midbrain. Extends from the caudal end of the posterior commissure rostrally to the superior medullary velum caudally. Bounded dorsally by superior and inferior colliculi, ventrally by DR, with boundaries defined by lines from mlf and cranial nerve nuclei to the cerebral aqueduct.

Abbreviations: ctg (Central Tegmental Tract), mlf (Medial Longitudinal Fasciculus), Mo5N (Motor Nucleus of the Trigeminal Nerve), PnO (Pontine Nucleus Oralis).

This table summarizes the anatomical landmarks and magnetic resonance imaging (MRI) signal characteristics used to manually delineate the seven brainstem nuclei of interest (PTg, LDTg, LDTgV, LC, SubC, DR, and PAG) on high-resolution 7-Tesla *ex vivo* T1-weighted images. Abbreviations used within the table are defined in the footnote.

Supplementary Table 5: Demographic and clinical characteristics of participants by institution.

	iRBD(NCNP)	iRBD(Juntendo)	iRBD(Kyoto)	iRBD(Osaka)	iRBD(Nagoya)	HA(NCNP)	HA(Kyoto)
Age (years)	69.6 ±5.9	72.2 ±4.4	73.3 ±5.1	70.0 ±4.6	71.9 ±6.0	68.9±5.8	68.9±5.7
Sex (male: female)	38:16	15:5	11:1	3:1	6:2	43:40	14:17
MoCA-J	24.9±3.0	23.3±3.5	25.1±1.9	25.5±3.3	25.5±4.5	24.8±2.2(estimated)	25.0±3.8(estimated)
MMSE	NA	NA	NA	NA	NA	28.7±1.1	28.6±1.9
SCOPA-AUT	8.1±3.7	12.0±6.1	9.8±2.9	13.8±4.6	11.9±4.4	NA	NA
MDS-UPDRS Part III	1.8±2.5	2.6±7.1	3.5±3.5	1.8±2.1	6.3±5.4	NA	NA
RBDSQ-J	7.6±3.0	6.7±3.1	6.8±1.7	10.8±2.5	7.6±3.9	NA	NA

This table provides a detailed breakdown of demographic data (age and sex) and key clinical scores (MoCA-J, MMSE, SCOPA-AUT, MDS-UPDRS Part III and RBDSQ-J) for both the healthy aged control (HA) and iRBD groups at each participating institution. All values are presented as mean ± standard deviation unless otherwise specified.

Abbreviations: NCNP, National Center of Neurology and Psychiatry; Juntendo Univ., Juntendo University; Kyoto Univ., Kyoto University; Osaka Univ., University of Osaka; Nagoya Univ., Nagoya University.

Supplementary Table 6: Lateralised ANCOVA analysis of brainstem nucleus volumes.

Nucleus	Side	HA (n=114)	iRBD (n=98)	Cohen's *d*	*t*	*p* (Bonferroni)
**PTg**	right	3.15 ± 0.92	2.86 ± 0.78	0.34	2.19	0.293
	**left**	**3.28 ± 1.02**	**2.85 ± 0.71**	**0.48**	**3.00**	**0.031***
LDTg	right	3.34 ± 0.61	3.11 ± 0.49	0.42	2.54	0.118
	left	2.68 ± 0.53	2.49 ± 0.40	0.40	2.48	0.139
**LDTgV**	**right**	**7.03 ± 1.48**	**6.43 ± 1.20**	**0.44**	**2.89**	**0.043***
	**left**	**6.73 ± 1.51**	**6.11 ± 1.11**	**0.47**	**3.09**	**0.023***
LC	right	8.25 ± 1.44	7.69 ± 1.15	0.43	2.58	0.106
	left	5.03 ± 0.99	4.66 ± 0.74	0.42	2.66	0.084
SubC	right	5.88 ± 1.76	5.42 ± 1.58	0.27	1.99	0.481
	left	3.98 ± 1.30	3.57 ± 1.07	0.34	2.32	0.212

Exploratory ANCOVA analysis of brainstem nucleus volumes separately for the left and right sides, adjusted for age, sex, and TIV. Bonferroni correction was applied across the 10 lateralised VOIs. * $p < 0.05$ (Bonferroni-corrected). Mean ± SD values are in mm^3.

Abbreviations: PTg, Pedunculopontine tegmental nucleus; LDTg, Laterodorsal tegmental nucleus; LDTgV, Laterodorsal tegmental nucleus, ventral part; LC, Locus coeruleus; SubC, Subcoeruleus nucleus

Supplementary Table 7: Partial correlations with PSG measures.

Nucleus	PSG variable	*r*	95% CI	*p*
**LDTgV**	**RWA/REM%**	**-0.491**	**[-0.767, -0.062]**	**0.028***
	Arousal Index	-0.031	[-0.467, 0.417]	0.896
	PLMI	0.064	[-0.390, 0.492]	0.790
	AHI	0.119	[-0.342, 0.533]	0.618
PTg	RWA/REM%	-0.379	[-0.703, 0.077]	0.100
	Arousal Index	0.156	[-0.308, 0.560]	0.512
	PLMI	-0.104	[-0.522, 0.355]	0.664
	AHI	-0.032	[-0.468, 0.417]	0.895

Partial Pearson correlations between adjusted grey matter volumes and polysomnographic measures, controlling for age, sex, and TIV. The 95% CIs were computed via Fisher's z transformation with degrees of freedom adjusted for the number of covariates. * $p < 0.05$ (uncorrected).

Abbreviations: LDTgV, Laterodorsal tegmental nucleus, ventral part; PTg, Pedunculopontine tegmental nucleus; PLMI, periodic limb movement index; AHI, apnoea–hypopnoea index.

Supplementary Figure 1: **Effect of ComBat harmonization on multi–site MRI data.**

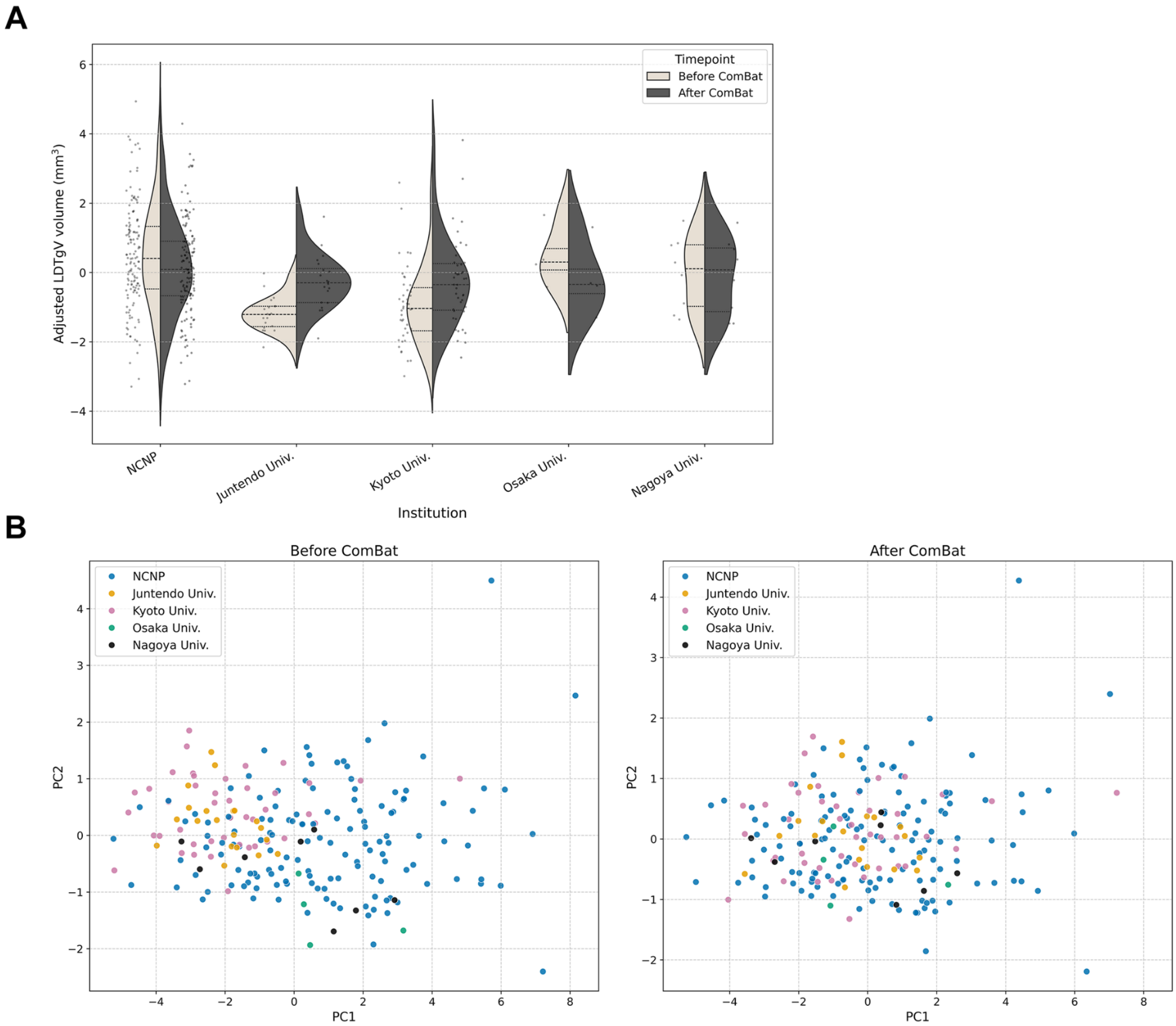

A. A split violin plot showing the distribution of effective volume for a representative VOI (LDTgV) across the sites. The left half of each violin (beige) shows the data distribution before ComBat, and the right half (dark grey) shows the one after ComBat. Individuals' data are shown as dots. The data distribution becomes comparable across the sites after ComBat harmonization.

B. Principal component analysis (PCA) of the volumes of the seven brainstem nuclei from all 212 participants (98 patients with isolated REM sleep behaviour disorder and 114 healthy aged controls). A single PCA was fitted to the pooled pre- and post-ComBat data. The eigenvalues of the first and second principal components were 5.89 and 0.70, explaining 84% and 10% of the total variance. Before ComBat (left panel), individuals' data tended to form a cluster following the sites. The clusters were not clear after ComBat (right panel), supporting successful removal of the site effect from the data structure.

Supplementary Figure 2: Brainstem nucleus volume overlapping with the VBM atrophy cluster

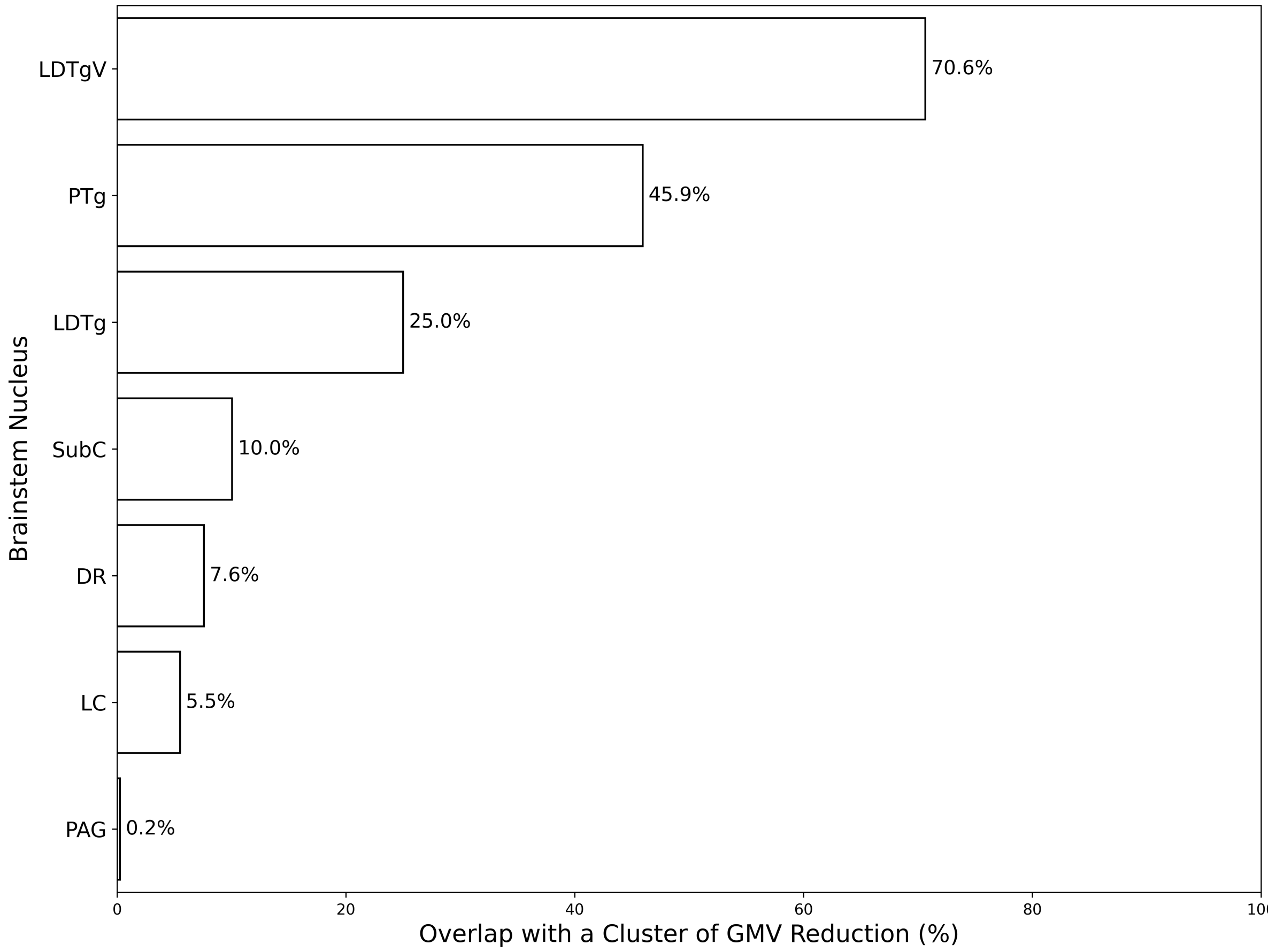

The *x*-axis represents the percentage of each nucleus volume contained within the VBM atrophy cluster in iRBD. Nuclei are sorted in descending order of the overlap percentage. The atrophic volume in iRBD primarily overlapped the LDTgV and PTg.

Abbreviations: GMV, grey matter volume; LDTgV, Laterodorsal tegmental nucleus, ventral part; PTg, Pedunculopontine tegmental nucleus; LDTg, Laterodorsal tegmental nucleus; SubC, Subcoeruleus nucleus; DR, Dorsal raphe nucleus; LC, Locus coeruleus; PAG, Periaqueductal grey; VBM, Voxel-based morphometry
